# Machined and plastic copings in three-element prostheses with different types of implantabutment joints: a strain gauge comparative analysis

**DOI:** 10.1590/S1678-77572010000300005

**Published:** 2010

**Authors:** Renato Sussumu NISHIOKA, Lea Nogueira Braulino de Melo NISHIOKA, Celina Wanderley ABREU, Luis Gustavo Oliveira de VASCONCELLOS, Ivan BALDUCCI

**Affiliations:** 1 DDS, MSc, PhD, Professor, Department of Dental Materials and Prosthodontics, São José dos Campos Dental School, São Paulo State University, São José dos Campos, SP, Brazil.; 2 Eng, MSc, Professor of Department of Mechanical Engineering-CETEC, São José dos Campos, Brazil.; 3 DDS, MSc, Graduate student, Department of Dental Materials and Prosthodontics, São José dos Campos Dental School, São Paulo State University, São José dos Campos, SP, Brazil.; 4 Eng, MSc, Professor of Department of Community Dentistry and Pediatric Clinic, São José dos Campos Dental School, São Paulo State University, São José dos Campos, SP, Brazil.

**Keywords:** Biomechanics, Dental implants, Dental prosthesis, Implant-supported dental prosthesis

## Abstract

**Objective:**

Using strain gauge (SG) analysis, the aim of this *in vitro* study was quantify the strain development during the fixation of three-unit screw implant-supported fixed partial dentures, varying the types of implant-abutment joints and the type of prosthetic coping. The hypotheses were that the type of hexagonal connection would generate different microstrains and the type of copings would produce similar microstrains after prosthetic screws had been tightened onto microunit abutments.

**Materials and methods:**

Three dental implants with external (EH) and internal (IH) hexagonal configurations were inserted into two polyurethane blocks. Microunit abutments were screwed onto their respective implant groups, applying a torque of 20 Ncm. Machined Co-Cr copings (M) and plastic prosthetic copings (P) were screwed onto the abutments, which received standard wax patterns. The wax patterns were cast in Co-Cr alloy (n=5), forming four groups: G1) EH/M; G2) EH/P; G3) IH/M and G4) IH/P. Four SGs were bonded onto the surface of the block tangentially to the implants, SG 1 mesially to implant 1, SG 2 and SG 3 mesially and distally to implant 2, respectively, and SG 4 distally to implant 3. The superstructure’s occlusal screws were tightened onto microunit abutments with 10 Ncm torque using a manual torque driver. The magnitude of microstrain on each SG was recorded in units of microstrain (µε). The data were analyzed statistically by ANOVA and Tukey’s test (p<0.05).

**Results:**

Microstrain values of each group were: G1= 338.1±223.0 µε; G2= 363.9±190.9 µε; G3= 415.1±53.5 µε; G4= 363.9±190.9 µε. No statistically significant difference was found between EH and IH, regardless of the type of copings (p>0.05). The hypotheses were partially accepted.

**Conclusions:**

It was concluded that the type of hexagonal connection and coping presented similar mechanical behavior under tightening conditions.

## INTRODUCTION

Osseointegrated dental implants have been a well-accepted and predictable treatment modality for rehabilitation of partially and completely edentulous patients. An implant-supported prosthesis may be under the influence of external (functional or parafunctional) and/or internal (preload) forces. The magnitude of this forces affects the amount of induced strains and stresses in all components of bone-implant-prosthesis complex^6,8-10,15,19,22,23,25,26^.

On tightening, the abutment screw exerts a compressive force to maintain the contact between the abutment and the implant surface. Due to the characteristics inherent to superstructure castings, component fit is not perfect but clinically acceptable. Torque of the prosthesis-abutment set induces stresses which are transmitted to the supporting bone.

Strain is defined as the ratio between the length of an object under stress and its original dimension; it is a dimensionless entity. Strain gauge (SG) is considered an indirect measurement that analyzes a physical effect, mechanical deformation, based on electrical measurements taken with a device called transducer. In short, it can be stated that deformations are normally imperceptible to the naked eye, so it is necessary a SG to measure them. SG is an electric sensor that quantifies a superficial deformation. Its working principle is based on the variation of the electrical resistance transformed into deformation levels.

The aim of this *in vitro* study was quantify the strain development during the fixation of three-unit screw implant-supported fixed partial dentures (FPDs), using SG analysis. The influence of types of implant-abutment joints (external and internal hexagon) and type of prosthetic coping (machined and plastic) was investigated.

The hypotheses were that type of hexagonal connection would generate different microstrains and type of copings would produce similar microstrains after prosthetic screws were tightened onto microunit abutments.

## MATERIALS AND METHODS

### Preparation of the test specimens

To simulate clinical conditions in a real-life arrangement, three external hexagon (3.75 mm diameter, 13 mm length; Master screw, Conexão Sistemas de Prótese, Arujá, SP, Brazil) and three internal hexagon type implants from mesial to distal: labeled A, B, and C (3.75 mm diameter, 13-mm depth; Conect AR; Conexão Sistemas de Prótese,) were arranged in the middle of two measurement model consisting of a 70x40x30 mm^3^ rectangular polyurethane block (Polyurethane F16, Axson, Cergy, France) with known mechanical properties (Young’s modulus of 3.6 GPa).

**Figure 1:**
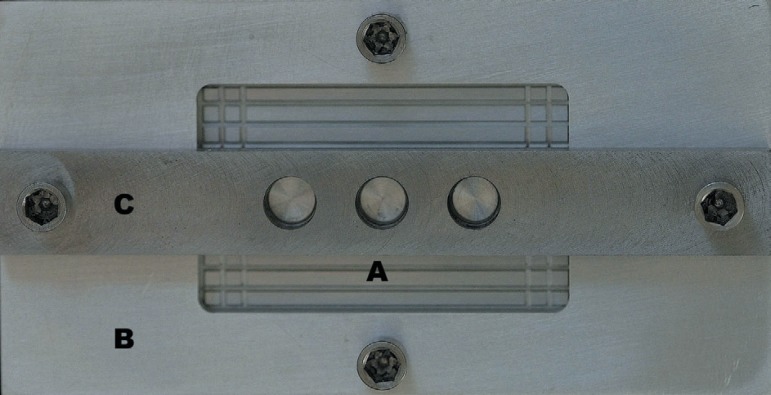
Aluminum matrix used to standardize in a straight line the implant placement in the polyurethane blocks and also to standardize the wax up of the superstructures. This matrix has lateral screws to keep the three components stable. A- component 1 (base); B- component 2; Ccomponent 3

A set of aluminum index consisting of three components was used to standardize in a straight line the implant placement into the polyurethane blocks and also to standardize the wax-up of superstructures (Figure 1). The implants were placed in the polyurethane block excluding strict asepsis.

**Figure 2:**
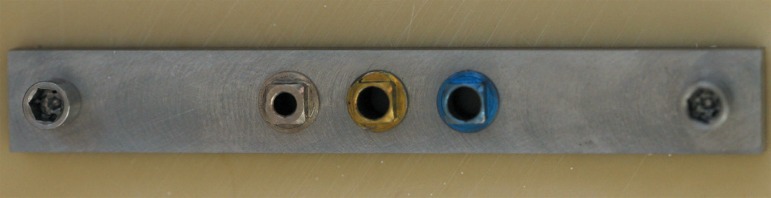
Component 3 with color-coded rings fixed onto the polyurethane block with horizontal screws to standardize locations for implant placement

Component 3 (the upper one), which determined the standardization of the distance and locations for implant placement, was fixed onto the polyurethane block with horizontal screws. Color-coded rings were screwed alternately into the three holes in component 3. The rings had progressively larger internal diameters compatible with standard twist drill used for implant placement (Drill guides; Conexão Sistemas de Prótese). The white ring was locations for implant placement compatible with the 2 mm, the yellow one with the 3 mm, and the blue one with the 3.15 mm twist drills. A handpiece with 16:1 reduction (16:1 handpiece; Kavo do Brasil, Joinville, SC, Brazil) was used to make the holes and insert the implants (Figure 2).

Three external hexagon (eH) (Master Screw, Conexão Sistemas de Prótese) and internal hexagon (IH) (Conect AR) implants measuring 3.75 mm in diameter and 13.00 mm in length were installed into the first and second polyurethane blocks, respectively. Microunit abutment types (Micro unit; Conexão Sistemas de Prótese) were screwed onto the implants to 20 Ncm torque using a manual torque driver (Torque driver, Conexão Sistemas de Prótese).

### Fabrication of metallic superstructures

All wax-up procedures (Kronen Wachs; Bego Bremer Goldschalgerei, Bremen, Germany) was standardized using component 1 (base) and component 2, which resulted in a rectangular compartment that allowed for the systematic reproduction of the wax-up of all the test specimens, especially in terms of thickness.

**Figure 3a and 3b:**
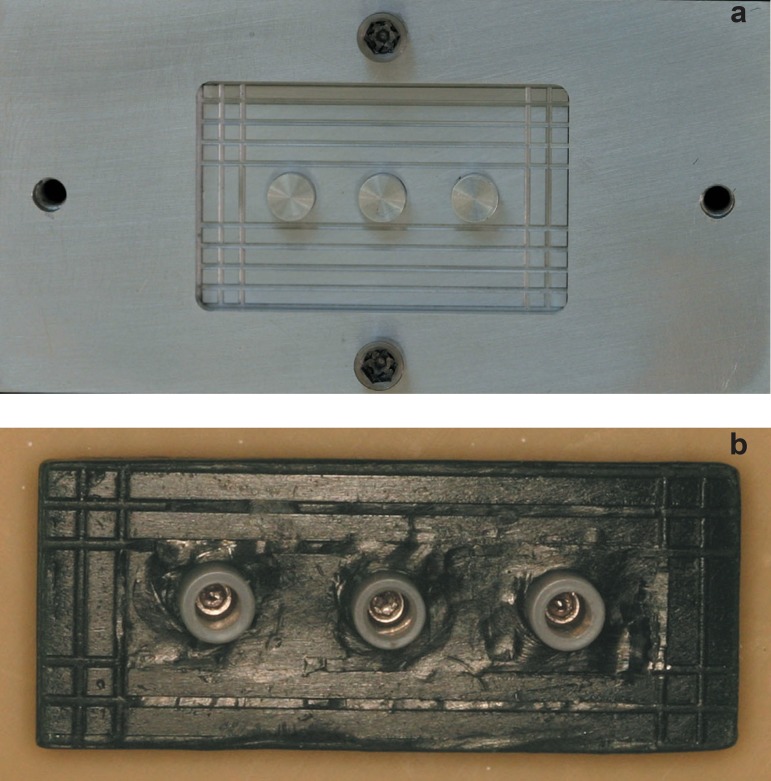
Component 1 and component 2 used for reproduction of the wax-up and wax patterns under polyurethane block

Each specific polyurethane block also served as the base for the abutment and wax-up procedures. Both plastic copings with metallic pre-machined Co-Cr collars (machined copings) and plastic copings were initially positioned directly on the abutment and the wax-up was adapted under slight pressure (Figure 3a and b).

The wax patterns with dimensions of 35x16x2 mm^3^ were sprued, invested and one-piece cast in a induction oven with cobalt-chromium alloy^5^ (Wirobond SG, Bego Bremer Goldschalgerei). To avoid bias resulting from manufacturing conditions, random sets comprising superstructures of different types were put together and cast. After removal from the investment material, the sprues were eliminated with the aid of carbide discs at low speed. The castings were airborne particle abraded with 110-μm particle aluminum oxide (Korox, Bego Bremer Goldschalgerei), under 60 psi pressure, care was taken not to damage the surface of coping and inspected under a binocular microscope for casting imperfections in the interior of each coping. The castings were then ultrasonically cleaned in isopropyl alcohol (Vitasonic II, Vita Zahnfabrick, Bad Säckingen, Germany) for 10 min and dried at room temperature.

**Figure 4:**
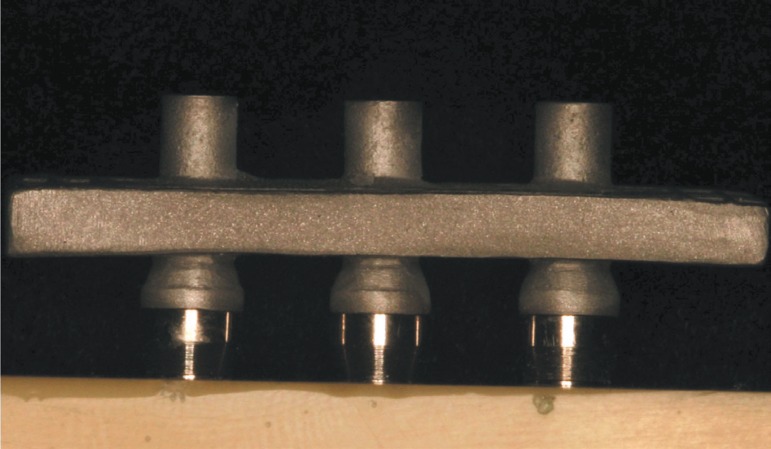
Fit and passivity of the superstructures

The superstructures were fit individually to their respective abutments and polyurethane blocks: stability of the set was checked without torque tightening. Superstructures showing signs of instability were excluded (Figure 4).

Each metallic structure was numbered and labeled according to its corresponding group. The whole sample was constituted of 20 superstructures distributed randomly and equally among the eH and IH groups. These were differentiated by casting of machined (M) and plastic (P) copings.

### SG analysis

For the exact determination of the sites for bonding the four SGs (KFG-02-120-c1-11N30C2, Kyowa electronic Instruments Co., Ltd, Tokyo, Japan), a line was drawn with a rule and a 0.7 mm pencil lead. The four SGs were centered along this line tangential to abutment. A thin film of methyl-2-cyanoacrylate resin (M-Bond 200; Vishay Measurements Group, Raleigh, NC, USA) was used to fix each SG, which was carefully positioned and held in place under slight pressure for three minutes. each gauge was wired separately and the four SGs were connected to a multichannel bridge amplifier to form one leg of the bridge.

**Figure 5:**
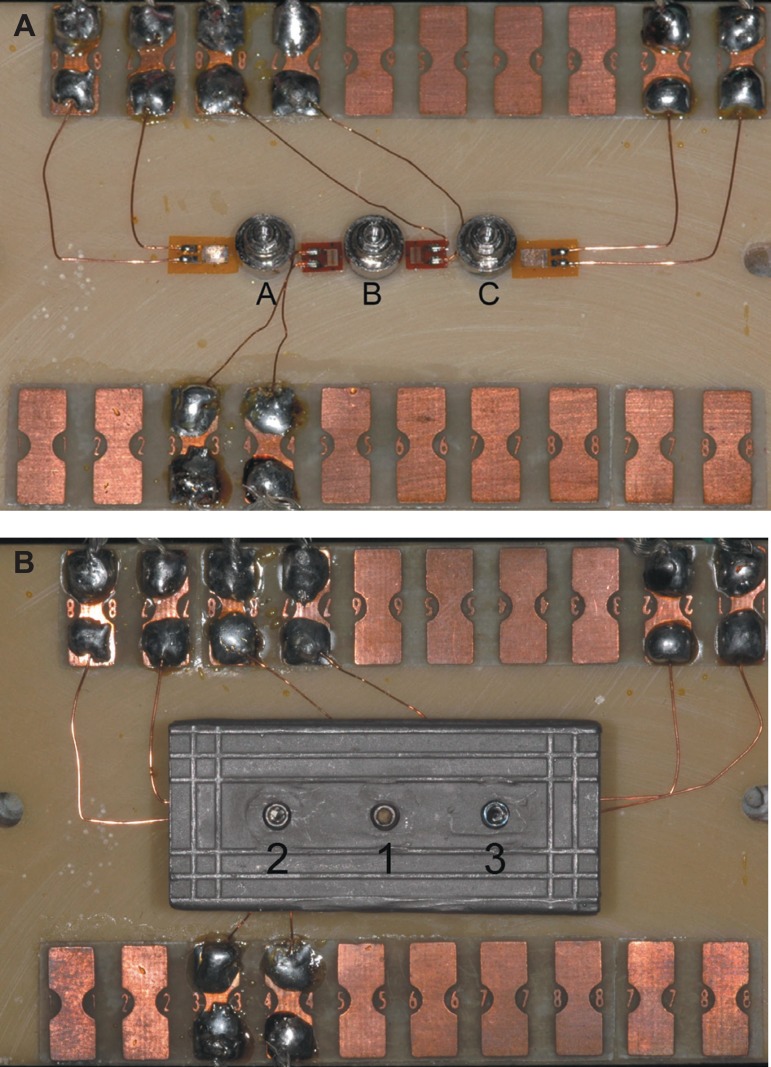
Strain gauges locations and the tightening sequence: first screw: implant B; second screw: implant A and third screw: implant C

All SGs were set to zero and then the superstructure was placed on the abutments. The superstructure’s occlusal screws were tightened onto the Microunit abutments using a hand-operated screwdriver, until the screws started to engage based on tactile sensation and with a torque of 10 Ncm using the manufacture’s manual torque-controlling device. each of the superstructures was screw tightened according the torque sequences with abutments: I) first screw: implant B (center), second: implant A and third screw: implant C and the sequence. The strains occurring were measured for the same duration (5 min). The screws were removed and the procedure was repeated other four times. A new set occlusal screw was used for each superstructure. The same investigator tightened all screws (Figure 5a and b).

The signals were interpreted, modified and processed using a computer program (Strain smart, Vishay Measurements Group). The data acquisition hardware (System 5000 Model 5100B; Vishay Measurements Group), which is an integrated system comprising an analog-digital converter, was used to condition the signals and the converter control and the connection to the computer.

The electrical variations were transformed arithmetically into microstrain units (με) by the data acquisition software installed in a microcomputer.

### Statistical analysis

The absolute values of strains were compared by two-way ANOVA followed by a post-hoc Tukey’s HSD test at 95 % confidence level (α=0.05). The absolute values of the four SGs were compared for this study, as the SGs are only capable of detecting stresses in a limited segment around the implants and do not clear statements as to whether compressive or tensile forces are present in a polyurethane area of a given magnitude.

## RESULTS

**Figure 6:**
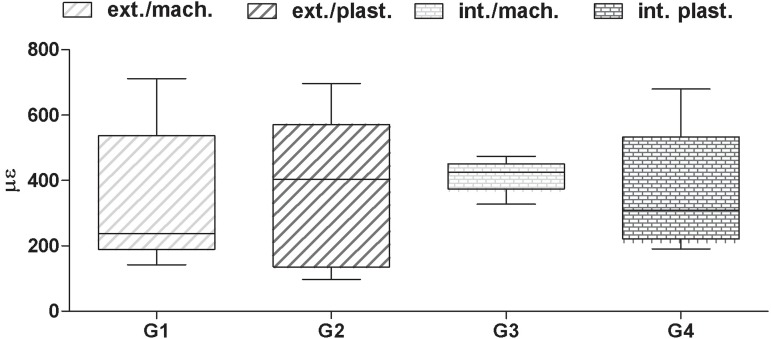
Dot plot for microdeformation (με) values obtained under the different experimental conditions

Figure 6 shows the microdeformation values (με) obtained after analysis of the mean microstrain values obtained by the four SGs positioned around the implant, for two types of prosthetic connection (EH and IH), as well as the type of coping (plastic and machined).

To evaluate the influence of tightening in relation to the type of prosthetic connection on the type of coping, in terms of microstrains, the data were subjected to two-way ANOVA after considering the distribution of the residuals. No statistically significant difference (p>0.05) was found between eH and IH, regardless of the type of copings. The hypotheses were partially accepted.

## DISCUSSION

To ensure the success of a surgical intervention, a factor that must be taken into account is the transfer of stresses and strains occurs around bone^3,8,9,10,25,26^. The reason for studying strains around implants is an attempt to define levels of safety, since there are studies reporting that an excessive load at the interface between the implant and the bone may be one of the causes of marginal bone loss^15^. The precise mechanism is not yet fully understood. Undoubtedly, there is a remodeling response around the bone under a given stress, or even in situations with absence of activity^11,27^. The use of SG, a well-suited design tool for analysis of the complex strain’s field around fixtures, is becoming more widespread^4,6,15,21,23^.

The present study used the SG analysis to quantify the strain development during the fixation of three-unit screw implant-supported FPDs, varying the types of implant-abutment joints (external and internal hexagon) and the type of prosthetic coping (plastic and machined).

The popularization of the use of plastic copings (without metallic collar) is directly attributable to a national trend for reducing costs. In the present study, the mean microstrain values recorded for eH and IH systems were similar, regardless of the type of coping used. This independence in the use of copings is consistent with the results reported by Karl, et al.^20^ (2005), who performed a study using the same number of fixations, although their prosthesis was built with five elements. Heckmann, et al.^13^ (2004) found no difference between these two types of copings. Previous SG studies have reported similar results^13,14,20^ , with fixed partial prostheses screwed onto implants, made from plastic or machined copings, producing the same magnitude of microdeformation during tightening of the retention screws, without any statistically significant difference between plastic and prefabricated copings before^13,14^ and after^20^ the application of a dental ceramic. Moreover, it should be noted that the care involved in handling multiple-element prostheses is very different from that involved in handling single-element ones, and the complexity of the laboratory procedures increases proportionally to the number of fixations involved. This may explain the results for single-element prosthesis reported by Carr, et al.^3^ (1991) and Byrne, et al.^2^ (1998), which evaluated gold machined copings.

The implant-abutment joint designs should have such junctions that reduce the peak bone interface stresses and strains^7^. In designs such as EH and IH, a compressive force is generated during the abutment screw tightening, which maintains the contact between the fixture-bearing surface and the bearing surface of the abutment. In EH, the abutment screw is the only element that keeps the fixture and the abutment assembled. Otherwise, in the IH, friction plays a crucial role in the maintenance of screw-joint in addition to the torque applied during abutment tightening. These fundamental differences in design affect the mechanical behaviors of implants^7,24^. From a prosthetic point of view, it would be important to assess bone deformation, more specifically bone strains near the bone/abutment/implant. In the present study, each specimen was screwed to the abutment using a same torque sequence. The values obtained with EH and IH showed no statistically significant differences. This finding suggests that the type of implant-abutment joint does not affect the magnitude of microdeformation in the fixation of three-unit screw implant-supported FPDs.

The one-piece casting method was chosen in order to eliminate several variables that would otherwise influence the analysis of the results, such as the material and transfer impression techniques^1,12^ , positioning of the analog to obtain a functional model and the welding techniques (standard or laser welding). The fabrication of one-piece casting method avoids the high risks of distortion when compared to structures that have to be cut and then welded. Welding may not improve the adaptation of three-element prostheses^28^.

However, one concern here is the misfit between the abutment and the prosthesis. The accuracy of metallic superstructures that adapt to the abutment has received undue attention and a rather unmerited concern, probably because it has always been dictated by the adaptation of conventional prostheses, and has been transferred incorrectly to implant-supported prostheses.

The cast structures showed satisfactory adaptation, which was confirmed by direct visualization together with tactile sensation using an explorer^18^. Çehreli, et al.^6^ (2004) reported a similar behavior in their evaluation. In the present study, it was not concerned with the occurrence of a gap, but with the seating of the test specimens on the abutment. Jemt and Book^16^ (1996) reported the extreme difficulty of visually checking for discrepancies of around 30 µm with the naked eye. Conventional laboratory procedures with the most diverse possibilities for the use of screwed or cemented copings are unable to produce metallic structures with passive adaptation^19^. Independently of the variables studied here, there was no occurrence of passivity during the tightening of the structures. This corroborates the studies of Assif, et al.^1^ (1996), who did not find a metallic structure with a design that could provide a passive adaptation, and is also consistent with the results of Jemt and Lie^17^ (1995), who reported the impossibility of connecting a multiple-element prosthesis on implants with a completely passive adaptation in a clinical situation.

## CONCLUSIONS

The SGs indicated that machined and plastic copings did not determine significant microstrains on three-element implant-supported prostheses. The external hexagon configuration showed similar values as those of the internal hexagon connection design.
